# Supplementary material for: Linking influenza epidemic onsets to covariates at different scales using a dynamical model
Source: PeerJ. 2018 Mar 8;6:e4440. doi: 10.7717/peerj.4440 (PMC5845579; doi:10.7717/peerj.4440)
Supplement: Supplemental Information 1 [file peerj-06-4440-s001.docx]

Web Appendices for “Linking influenza epidemic onsets to covariates at different scales using survival analysis models” by Roussel et al.

WEB MATERIAL 1: Variability of methods and data

The primary source of variability between studies concerns the data used to follow the spread of influenza and the metric used to estimate epidemic onsets (or proxy) from the data. Regarding data, many studies were based either on influenza-related mortality (1-3) or on influenza syndromic cases derived from general practitioner networks (4-6), which are easier to collect than laboratory confirmed influenza cases (7, 8).

About metrics, two are generally used: the onset and the peak (1, 8, 9). The epidemic onset is obviously the best option but it is complex to measure, and no consensual definition exists to estimate it (e.g., definition of a threshold - (2, 9); date on which the number of cumulative cases reached 25% of the total cases reported - (10)). Conversely the peak is easier to observe but it is less accurate because the timing difference between the onset and the peak can vary according to areas and years. In addition, covariates such as the mobility of infected people, which may vary between the onset and the peak, may have different impact on the peak and on the onset.

WEB MATERIAL 2: Data

1. Description of epidemiological data

Epidemiological data come from the Réseau des GROG surveillance network, which is a French surveillance network made up of general practitioners (GP) and pediatricians. These physician sentinels identify cases of respiratory pathogens including influenza. Each region has on average 25 physician sentinels (from 10 to 75 depending on regions and epidemic years) involved in the GROG network. Every week from October to April they report the intensity of their activity by informing the number of days they worked, the number of medical acts performed and the number of acute respiratory infection (ARI) defined as the sudden onset of at least one respiratory sign (cough, rhinitis, coryza, etc.) and at least one systemic sign suggesting an acute infectious context (fever, fatigue, headache, myalgia, malaise, etc.). In addition, sentinels randomly realize nasal/pharyngeal swab samples on patients with a less than 48 hours ARI, allowing virological confirmation (or rejection) of influenza infections. Using the weekly information reported by each physician sentinel (clinical reports and virological samples analysis), the GROG network is able to provide an estimation of the number of influenza-infected individuals called the influenza incidence.

2. Calculation of the incidence

First the ARI incidence (*I_ARI_*) for a region and a week *t* is defined as:

$I_{ARI}\left( t \right)=\left( \frac{{Ped}_{R}}{{Ped}_{GROG participants}(t)}\cdot{ARI}_{Ped}\left( t \right) \right)+ \left( \frac{{GP}_{R}}{{GP}_{GROG participants}(t)}\cdot{ARI}_{GP}(t) \right)$

where *ARI_GP_(t)* and *ARI_PED_(t)* stand for the number of ARI cases, for week *t*, respectively, reported by GP and pediatricians from the GROG network. *GP_R_* and *Ped_R_* are, respectively, the number of GP and pediatricians of a region. *GP_GROG participants_(t)* and *Ped_GROG participants_(t)*, respectively, represent the number of GP and pediatricians who participated in surveillance the week *t*. Age of infected individuals was not taken into account assuming that climatic covariates have a uniform impact on influenza spread within the population.

Second, the GROG network estimates influenza incidence relying on both the ARI incidence and virological data. For each week of each region, an influenza positivity rate (for all circulating strains) is defined as the ratio of the number of positive samples on the total number of samples collected over a week. It is calculated using a moving average of order 3 taking into account the positive rate of the week concerned and the ones before and after in order to remove excessive fluctuations. We assumed that the positive rate corresponds to the actual proportion of influenza cases among ARI cases reported by the GROG network. The influenza incidence (*I_influenza_*) is defined as the ARI incidence weighted by the positivity rate (*T_+_*):

$$I_{influenza}\left( t \right)=I_{ARI}\left( t \right) \cdot T_{+}(t)$$

WEB MATERIAL 3: Including or excluding non-epidemic data points?

A non-epidemic data point corresponds to an observation (in a given epidemic year of given region) for which no epidemic occurred over the entire study period.

On a technical point of view, the Matlab program can deal with these points (the associated likelihood is simply given by $e^{-\sum_{i=0}^{Tmax-1} \lambda\left( R,Y,i \right)}$, *Tmax* being the last week of the survey). So choosing to include or exclude them from the analysis should only be motivated by the way they affect the analysis.

*What causes them?*

As it is built, the model is probabilistic. It quantifies, for each year, each region and each week, the probability that an epidemic occurs. Epidemics may occur or not every week for every year in every region. So it is possible that no epidemic occurs at all in a region during a given epidemic year just because of chance.

However, what the model does not take into account is the fact that some regions might be “resistant” (or at least much more resistant than the others) to influenza epidemics. This could be the case, for example, if a sufficient proportion of individuals is immune to the virus (e.g., have been exposed during past epidemic years to a variant that is close to the one circulating this year).

It is important to make a distinction between these two scenarios. The first one (stochastic effects) is handled naturally by the model. It assumes that non-epidemic data points work in the same way as epidemic ones, but had a qualitatively different outcome because of chance. In the second one (regions resistant to influenza epidemics), the non-epidemic data points obey a different logic than the epidemic ones and they should be excluded from the analysis (or at least treated separately).

*How can we make the distinction?*

The second situation presented earlier might be a bit extreme, but an important question still holds: can non-epidemic data points be considered as obeying the same mathematical logic as epidemic ones? Can we add them in the analysis because they are in the direct continuity of epidemic data points? Contrarily, do they contain a different type of information?

One way to investigate this question is to evaluate, for each of these five data points, if the model inferred from the analysis that excludes them is able to predict their non-epidemic observed outcome. Concretely, we followed the following steps:

Step 1: let us consider a sub-model linking influenza epidemic onset dates to a set of covariates. Let us consider the corresponding parameters inferred from the analysis of the data set excluding non-epidemic data points (typically those presented in WEB TABLE 1). For each of the five non-epidemic data points, we use the equation linking *λ* to model parameters and covariates to estimate the associated epidemic onset rate for every week of the study period.

Step 2: we calculate the probability of observing no epidemic for each of the five data points ($P\left( no epidemic \right)=e^{-\sum_{i=0}^{Tmax-1} \lambda\left( R,Y,i \right)}$).

To understand what happens, let us consider the example of the four sub-models linking epidemic onset dates to temperature. In WEB TABLE 2 we reported the decimal logarithm of the probability of observing no epidemic for the five non-epidemic data points.

A priori, there is no reason why these probabilities should be close to one, but at least they should not be too small. A couple of percent should be enough because non-epidemic observations occur in only around 5% of the observed data points.

What we found was far from that. In this example, probabilities were all very low (<10^-3^, very often <10^-5^ and quite often <10^-10^, see WEB TABLE 2). This means that with the model parameters inferred from the data set excluding non-epidemic data points, it was virtually impossible to explain why no epidemic occurred in the five regions-years.

This rapid analysis does not provide a rigorous proof that non-epidemic data points contain different information but helps to understand what we will show below. However, from these rapid calculations, we think that one should be suspicious about the relevance of including non-epidemic data points in the analysis. Is it really worth it? What does that change in the analysis?

*Implications for the analysis*

If the Matlab program was as a person, what he would want would be to minimize the model deviance. And non-epidemic data points provide an interesting target to gain easy deviance points. Let's imagine that the program manages to increase the probability of observing no epidemic (for any of the five non-epidemic data points) from 10^-10^ to 10^-3^. That represents a gain of more than 32 points in deviance!

As a consequence, regarding the inference of the model parameters, what the program will do is to find a combination of model parameters that explains non-epidemic data points with a reasonable probability. We can see that clearly in WEB TABLE 3, where we show the (decimal logarithm of the) same probabilities we calculated earlier but evaluated with the model parameters inferred from the analysis including the five non-epidemic data points. We now obtain probabilities that are above 10^-3^ for all the sub-models considered in all the five non-epidemic regions-years. Of course this imposes a strong constraint on the model parameters’ estimates.

Another interesting thing to look at is the consequence of adding non-epidemic data point in the analysis on the test of the link between epidemic onset dates and covariates (WEB TABLE 4). First we can see on that table that parameter estimates are now quite different, illustrating what we said earlier. Sometimes including non-epidemic years will lead to higher absolute estimated values, sometimes to lower ones, and sometimes estimates that go in the opposite direction (T: annual and H: spatiotemporal).

We can also see that P values are globally higher so that now no association is found to be significant. Contrarily to what one could imagine, this is not because the absolute value of the estimated parameters nor the observed LRT statistics are smaller, but mainly because rejection thresholds are higher. One possible explanation for that would be that, by trying to fit both epidemic and non-epidemic data points, the model ends up explaining both poorly, leaving important opportunities for additional random variables to improve the quality of the model fit to data.

To summarize, we think that including non-epidemic data points is a risky move when the absence of an observed epidemic does not appear to be in the continuity of what could be expected from simple stochastic phenomena. It can have diverse undesirable effects including: i) affecting the estimation of the model parameters in a way that goes beyond the simple add up of new consistent points in the data set and ii) increasing the rejection threshold, leading to a loss of statistical power when testing for the links between the response variable and covariates.

As a result, non-epidemic data points should be included with caution. In the case where there is an important suspicion that they are not in the direct continuity of epidemic data points, we recommend to exclude them from the analysis. In such a situation, keeping them could also be an option, but we strongly recommend to adopt an approach inspired from zero inflated models where an outcome (here the absence of an epidemic) of the base model (here the dynamical model) would be considered to be potentially more likely to occur than what is expected from the base model.

WEB FIGURES

Web Figure 1 – Incidence per 100000 individuals per region according to studied seasons from week 41 of a year to week 14 of the year after.

Web Figure 2 - Map of France showing the geographical locations of the 125 meteorological stations (in black) of the 19 studied French regions.


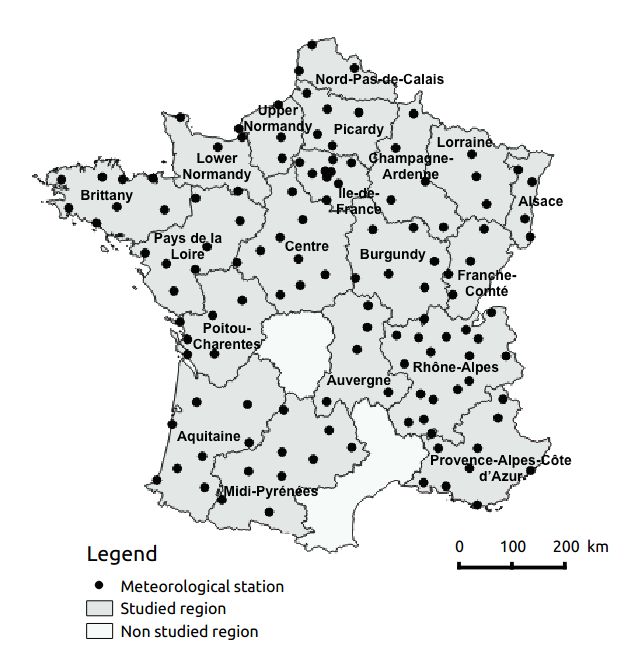


Web Figure 3 - Theoretical distributions under the null hypothesis with the threshold (the 95^th^ quantile) in black and the observed LRT (in red) for each sub-covariate of the temperature covariate estimated from eighteen French regions and six epidemic years. a) sub-covariate TW; b) TR; c) TY and d) Tres. For comparison, we presented the 95% rejection threshold that would be used with the chi-square approximation (dashed lines)


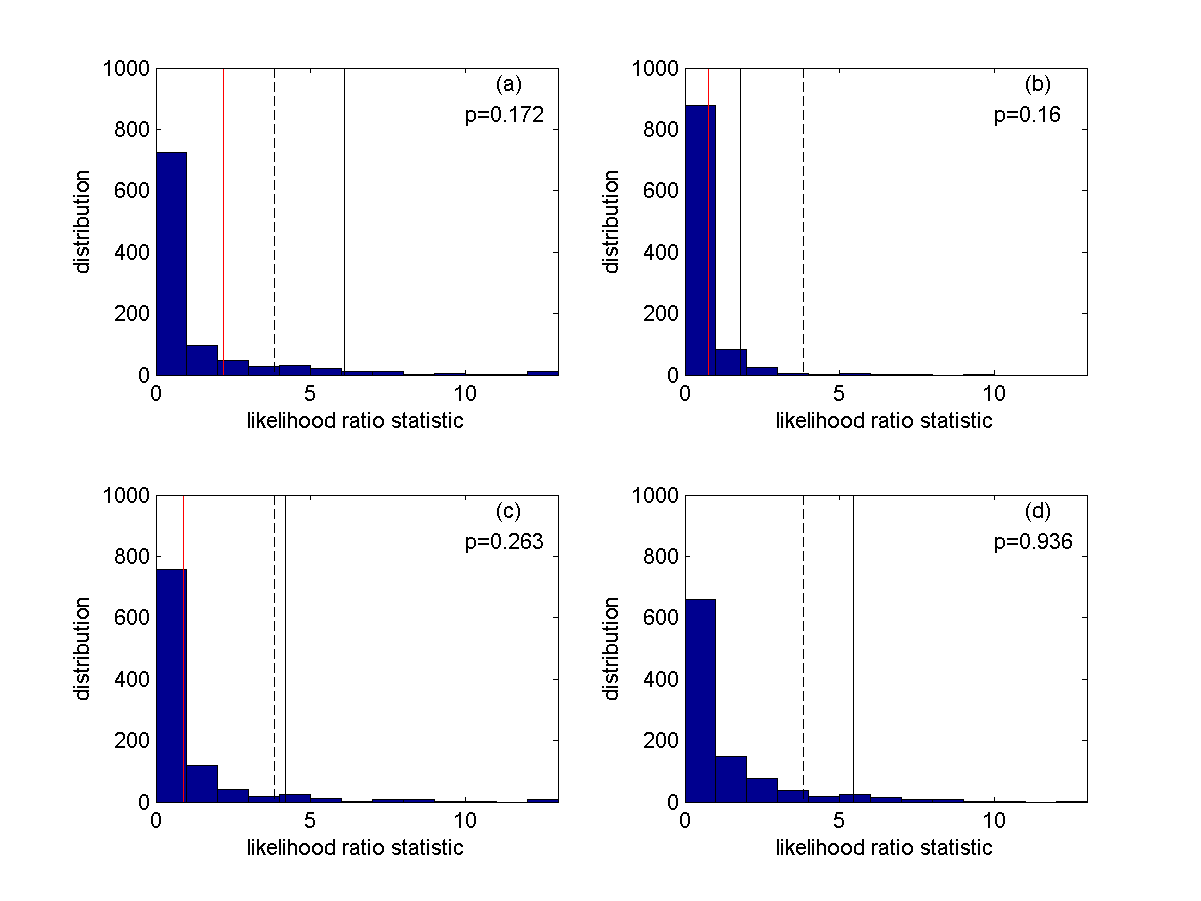


Web Figure 4 - Theoretical distributions under the null hypothesis with the threshold (the 95^th^ quantile) in black and the observed LRT (in red) for each sub-covariate of the absolute humidity covariate estimated from eighteen French regions and six epidemic years. a) sub-covariate HW; b) HR; c) HY and d) Hres. For comparison, we presented the 95% rejection threshold that would be used with the chi-square approximation (dashed lines)


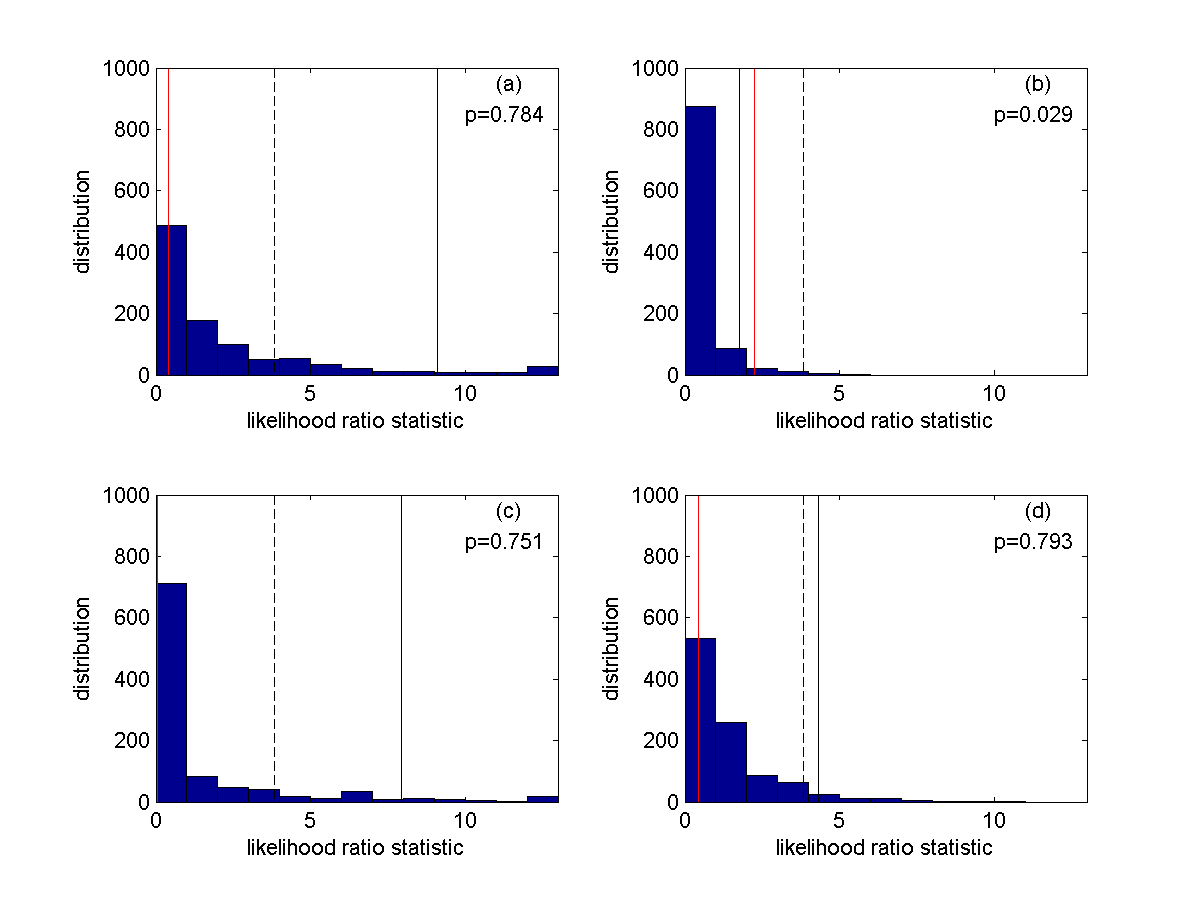


Web Figure 5 - Theoretical distributions under the null hypothesis with the threshold (the 95^th^ quantile) in black and the observed deviance (in red) of the covariate related mobility flows estimated from eighteen French regions and six epidemic years. (a) Corrected model and (b) uncorrected model.


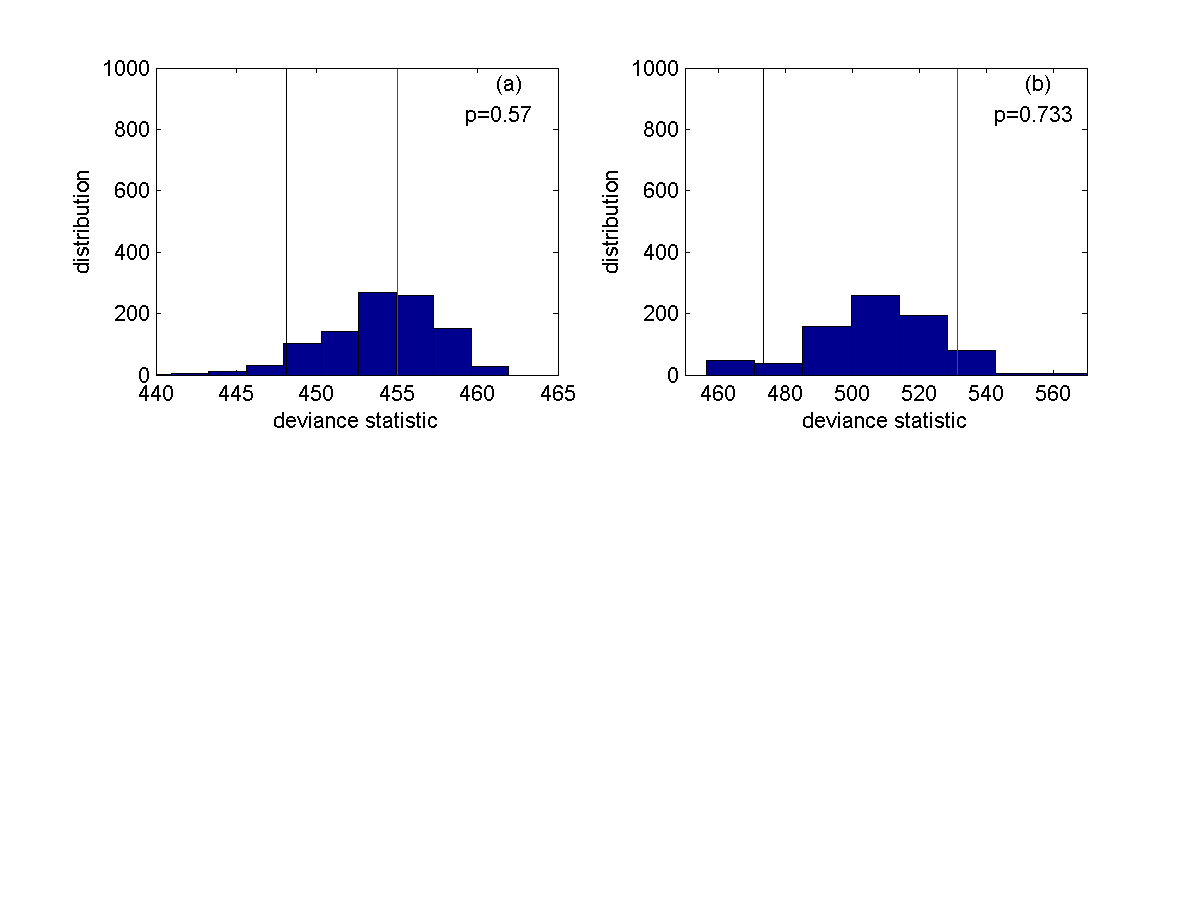


Web Figure 6 - Theoretical distributions under the null hypothesis with the threshold (the 95^th^ quantile) in black and the observed LRT (in red) of the covariates associated to demographic factors estimated from eighteen French regions and six epidemic years. a) Population size and b) proportion of children. For comparison, we presented the 95% rejection threshold that would be used with the chi-square approximation (dashed lines)


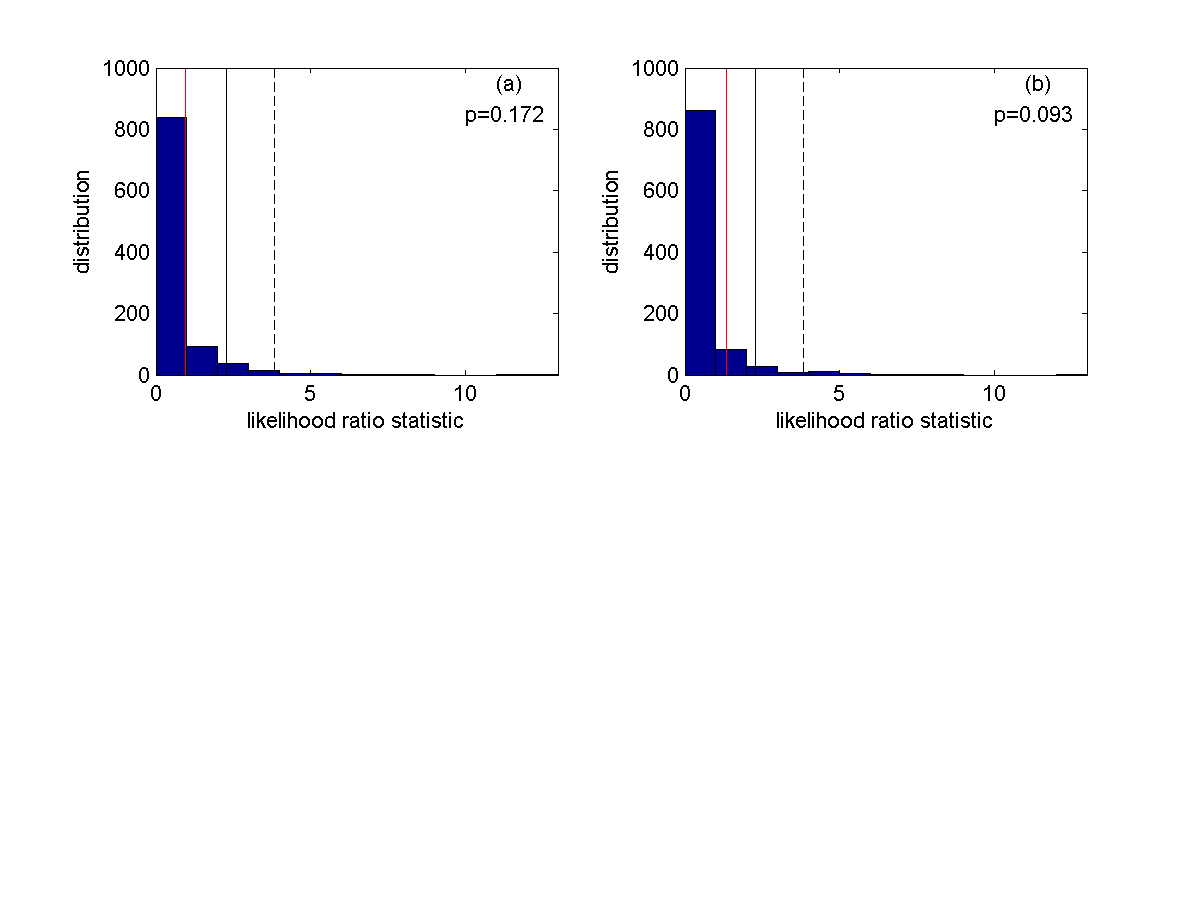


WEB TABLES

Web Table 1 - Estimates of parameters for climatic covariates.

| Parameter | Covariate tested | | | | | | | | | | | |
| --- | --- | --- | --- | --- | --- | --- | --- | --- | --- | --- | --- | --- |
|  | Mobility flow | | Demographic | | Humidity | | | | Temperature | | | |
|  | corrected | Un-corrected | Population size | Proportion of children | Global | spatial | Inter-annual | Spatio  temporal | Global | spatial | Inter-annual | Spatio  temporal |
| *Log(α)* | -0.017 | -0.674 | -0.0729 | -0.067 | -0.044 | -0.0786 | -0.0196 | -0.0503 | -0.078 | -0.081 | -0.027 | -0.068 |
| *Log(c)* | 12.35 | -1.99 | 12.77 | 12.74 | 12.380 | 12.813 | 12.497 | 12.473 | 13.01 | 12.89 | 12.60 | 12.61 |
| *a_S_* | 0.024 | - | 0.12 | - | 0.133 | - | 0.160 | 0.084 | 1.439 | - | -0.596 | 0.088 |
| *a_C_* | 0.496 | - | - | 0.121 | 0.150 | - | 0.398 | 0.186 | -1.197 | - | 1.026 | 0.189 |
| *a_HW_* | -0.029 | - | 3.94 | 3.853 | -0.020 | 5.276 | 0.019 | 0.726 | - | 4.747 | -2.468 | -0.174 |
| *a_HR_* | -3.074 | - | - | - | -0.734 | -0.476 | -2.211 | -0.994 | -2.079 | - | -17.41 | -0.933 |
| *a_HY_* | 5.767 | - | 2.88 | 2.968 | 2.427 | 3.007 | -0.045 | 2.976 | 5.523 | 2.606 | - | 2.814 |
| *a_Hres_* | -0.999 | - | 0.007 | 0.142 | -0.113 | -0.300 | -1.671 | -0.300 | -1.822 | -0.315 | -4.176 | - |
| *a_TW_* | -0.493 | - | -3.88 | -3.769 | - | -5.174 | -0.092 | -0.841 | -0.493 | -4.592 | 1.880 | -0.018 |
| *a_TR_* | 1.949 | - | - | - | 0.244 | - | 1.252 | 0.403 | -1.077 | -0.256 | 2.342 | 0.385 |
| *a_TY_* | -7.360 | - | -3.21 | -3.375 | -2.785 | -3.395 | - | -3.434 | -7.425 | -2.931 | -0.384 | -3.240 |
| *a_Tres_* | -0.738 | - | 0.218 | -0.081 | 0.044 | 0.200 | 1.473 | - | -0.209 | 0.272 | 2.982 | 0.046 |
| *a_0_* | -10.42 | -4.68 | -10.35 | -10.35 | -9.556 | -10.55 | -10.38 | -9.727 | -11.95 | -10.50 | -15.73 | -9.55 |

Web Table 2: decimal logarithm of the probability of observing no epidemic in the five non-epidemic data points estimated with parameters inferred from the analysis excluding non-epidemic data points. Results are shown only for the four sub-models used for testing the link between epidemic onset dates and temperature.

| Data point = Region (epidemic year) | Sub-model | | | |
| --- | --- | --- | --- | --- |
|  | T: global | T: spatial | T: annual | T: spatiotemporal |
| Haute-Normandie (2007-2008) | -8.58 | -4.93 | -6.08 | -7.57 |
| Provence-Alpes-Côte-D’azur (2007-2008) | -14.46 | -5.02 | -25.65 | -13.28 |
| Auvergne (2011-2012) | -10.86 | -5.78 | -4.25 | -13.88 |
| Haute-Normandie (2011-2012) | -12.82 | -8.35 | -5.00 | -16.46 |
| Pays-de-la-Loire (2011-2012) | -13.59 | -5.89 | -5.81 | -14.28 |

Web Table 3: decimal logarithm of the probability of observing no epidemic in the five non-epidemic data points estimated with parameters inferred from the analysis including non-epidemic data points. Results are shown only for the four sub-models used for testing the link between epidemic onset dates and temperature.

| Data point = Region (epidemic year) | Sub-model | | | |
| --- | --- | --- | --- | --- |
|  | T: global | T: spatial | T: annual | T: spatiotemporal |
| Haute-Normandie (2007-2008) | -2.00 | -2.34 | -2.26 | -1.98 |
| Provence-Alpes-Côte-D’azur (2007-2008) | -1.60 | -2.52 | -1.78 | -1.55 |
| Auvergne (2011-2012) | -2.71 | -1.39 | -1.34 | -1.49 |
| Haute-Normandie (2011-2012) | -1.85 | -1.40 | -1.01 | -1.10 |
| Pays-de-la-Loire (2011-2012) | -3.27 | -1.58 | -1.60 | -1.61 |

Web Table 4: differences in estimates of the parameter of interest, observed LRT, 95% thresholds of the LRT obtained from the permutation procedure and P values between the analysis where non-epidemic years were included and excluded. Results are presented for the four components of the two climatic covariates (temperature, T; humidity, H) and the two demographic covariates (population size and the proportion of children, prop. children)

|  | estimate | | Observed LRT | | 95% threshold (LRT) | | P value | |
| --- | --- | --- | --- | --- | --- | --- | --- | --- |
|  | excluded | included | excluded | included | excluded | included | excluded | included |
| T: global | -0.4932 | -0.3915 | 0.1870 | 3.8622 | 6.0798 | 20.9497 | 0.1718 | 0.4356 |
| T: spatial | -0.2557 | -0.4346 | 0.7752 | 1.3997 | 1.8026 | 6.8800 | 0.1598 | 0.4366 |
| T: annual | -0.3841 | 0.1906 | 0.8816 | 1.2058 | 4.1795 | 8.5556 | 0.2627 | 0.4665 |
| T: spatiotemporal | 0.0461 | 0.2987 | 0.0202 | 0.5286 | 5.4401 | 3.6685 | 0.9361 | 0.4605 |
| H: global | -0.0200 | -0.1131 | 0.3981 | 0.3187 | 9.0969 | 20.6519 | 0.7842 | 0.8232 |
| H: spatial | -0.4763 | -0.1322 | 2.2409 | 0.1902 | 1.7614 | 6.2979 | 0.0290 | 0.7902 |
| H: annual | -0.0449 | 0.2738 | 0.0505 | 2.9623 | 7.9286 | 9.7823 | 0.7512 | 0.2977 |
| H: spatiotemporal | -0.3004 | 0.1990 | 0.4509 | 0.2055 | 4.3136 | 2.9695 | 0.7932 | 0.6294 |
| Population size | 0.1274 | 0.1488 | 0.9521 | 1.8024 | 2.2829 | 6.5359 | 0.1718 | 0.3397 |
| Prop. children | 0.1215 | 0.0297 | 1.3576 | 0.0792 | 2.2672 | 6.3188 | 0.0929 | 0.8472 |

REFERENCES

1. Viboud C, Bjørnstad ON, Smith DL *et al*: Synchrony, Waves, and Spatial Hierarchies in the Spread of Influenza. *Science.* 2006;312(5772):447-451.

2. Shaman J, Pitzer VE, Viboud C *et al*: Absolute Humidity and the Seasonal Onset of Influenza in the Continental United States. *PLoS Biol.* 2010;8(2):e1000316.

3. Alonso WJ, Viboud C, Simonsen L *et al*: Seasonality of Influenza in Brazil: A Traveling Wave from the Amazon to the Subtropics. *Am J Epidemiol.* 2007;165(12):1434-1442.

4. Charaudeau S, Pakdaman K, Boëlle P-Y: Commuter Mobility and the Spread of Infectious Diseases: Application to Influenza in France. *PLoS ONE.* 2014;9(1):e83002.

5. Crépey P, Barthélemy M: Detecting Robust Patterns in the Spread of Epidemics: A Case Study of Influenza in the United States and France. *Am J Epidemiol.* 2007;166(11):1244-1251.

6. Huppert A, Barnea O, Katriel G *et al*: Modeling and Statistical Analysis of the Spatio-Temporal Patterns of Seasonal Influenza in Israel. *PLoS ONE.* 2012;7(10):e45107.

7. Stark JH, Cummings DAT, Ermentrout B *et al*: Local Variations in Spatial Synchrony of Influenza Epidemics. *PLoS ONE.* 2012;7(8):e43528.

8. Yu H, Alonso WJ, Feng L *et al*: Characterization of Regional Influenza Seasonality Patterns in China and Implications for Vaccination Strategies: Spatio-Temporal Modeling of Surveillance Data. *PLoS Medicine.* 2013;10(11):e1001552.

9. Gog JR, Ballesteros S, Viboud C *et al*: Spatial Transmission of 2009 Pandemic Influenza in the US. *PLoS Comp Biol.* 2014;10(6):e1003635.

10. He D, Dushoff J, Eftimie R *et al*: Patterns of spread of influenza A in Canada. *Proceedings of the Royal Society of London B: Biological Sciences.* 2013;280(1770).
